# Favourable long-term recovery after decompressive craniectomy: the Northern Finland experience with a predominantly adolescent patient cohort

**DOI:** 10.1007/s00381-022-05568-7

**Published:** 2022-06-24

**Authors:** Tommi K. Korhonen, Maria Suo-Palosaari, Willy Serlo, Maija J. Lahtinen, Sami Tetri, Niina Salokorpi

**Affiliations:** 1 Department of Neurosurgery, Oulu University Hospital, Neurocenter, Oulu, Finland; 2 Research Unit of Clinical Neuroscience, MRC Oulu, Oulu University Hospital and University of Oulu, Oulu, Finland; 3 Department of Diagnostic Radiology, Physics and Technology, Research Unit of Medical Imaging, Oulu University Hospital and University of Oulu, Oulu, Finland; 4 PEDEGO Research Unit, MRC Oulu, and Department of Children and Adolescents, University of Oulu, Oulu University Hospital, Oulu, Finland

**Keywords:** Brain injury, Complications, Outcome, Trauma

## Abstract

**Purpose:**

Decompressive craniectomy (DC) is an effective treatment of intracranial hypertension. Correspondingly, the procedure is increasingly utilised worldwide. The number of patients rendered vegetative following surgery has been a concern—a matter especially important in children, due to long anticipated lifetime. Here, we report the long-term outcomes of all paediatric DC patients from an 11-year period in a tertiary-level centre that geographically serves half of Finland.

**Methods:**

We identified all patients younger than 18 years who underwent DC in the Oulu University Hospital between the years 2009 and 2019. Outcomes and clinicoradiological variables were extracted from the patient records.

**Results:**

Mean yearly prevalence of brain injury requiring DC was 1.34/100 000 children—twenty-four patients underwent DC during the study period and 21 (88%) survived. The median age of the patients was 16.0 years, and the median preoperative GCS was 5.0 (IQR 5.0). Fifteen patients (63%) had made a good recovery (Extended Glasgow Outcome Scale ≥ 7). Of the surviving patients, two (9.5%) had not returned to school. After traumatic brain injury (*n* = 20), the Rotterdam CT score (mean 3.0, range 1 to 5) was not associated with mortality, poor recovery or inability to continue school (*p* = 0.13, *p* = 0.41, *p* = 0.43, respectively). Absent basal cisterns were associated with mortality (*p* = 0.005), but not with poor recovery if the patient survived DC (*p* = 0.81). Hydrocephalus was associated with poor recovery and inability to continue school (*p* = 0.01 and *p* = 0.03, respectively).

**Conclusion:**

Most of our patients made a favourable recovery and were able to continue school. No late mortality was observed. Thus, even in clinically and radiologically severely brain-injured children, decompressive craniectomy appears to yield favourable outcomes.

**Supplementary information:**

The online version contains supplementary material available at 10.1007/s00381-022-05568-7.

## Introduction

In the wake of the recent rigorous clinical trials [1, 2], decompressive craniectomy (DC) is becoming a key treatment modality in modern neurosurgery. Concerning paediatric patients, traumatic brain injury (TBI), commonly present with diffuse brain oedema, is the most common aetiology of intractably increased intracranial pressure (ICP) requiring DC [3]. According to a recent systematic review on paediatric patients, DC effectively controls ICP following TBI, and may decrease mortality and improve recovery compared to medical treatment, though the level of evidence is very low and studies with long-term follow-up and data on recovery are lacking [4].

Both craniectomy and later cranioplasty are associated with a high incidence of complications. Complications following DC are similar in adults and children. Complications mainly comprise surgical site infections (SSI), cerebrospinal fluid (CSF) disorders and haemorrhages [5], but data on the incidence of complications following paediatric DC is scarce. Furthermore, the paediatric population is especially burdened by cranioplasty complications such as bone flap resorption [6, 7]. Few risk factors that predispose paediatric patients to DC complications or poor recovery have been established. In order to study these outcome predictors, large patient series are needed, which is difficult to achieve in paediatric neurosurgical patients. Commendably, a trend towards multicentre and meta-analytical approaches is emerging [8–11].

In the present study, we report the long-term outcomes and recovery predictors of paediatric DC patients of the Oulu University Hospital from an 11-year period. The Oulu University hospital is a tertiary-level centre with a catchment area of 51% of the total area of Finland and a catchment population of 740 000 inhabitants. Acute neurosurgical care in the Northern Finland is provided only in the Oulu University Hospital. Thus, the presented data is an accurate epidemiological estimate of the incidence of very severe paediatric brain injury.

## Methods

### Patients, follow-up and clinical data

We identified all patients younger than 18 years of age who underwent DC for intractably increased ICP between the years 2009 and 2019 at the Oulu University Hospital. Customary to the Finnish healthcare system, all neurosurgical services in the catchment area are provided by the Oulu University Hospital and all patients from the catchment area with neurosurgical complications are also admitted to the Oulu University Hospital. The present study provides robust follow-up data with epidemiological importance.

All the patients (*n* = 24) were followed from the primary insult until the last patient record evaluation done on the 10th of February 2020 or their death if they passed away earlier. Craniectomies conducted for other indications than alleviation of intracranial hypertension were excluded from the present study. Data on patient characteristics, operative technique, timing, reoperations and complications were collected from the patient records. Radiological bone flap resorption after autologous cranioplasty was evaluated using the Oulu resorption score [12].

For TBI patients, the severity of the primary insult was evaluated by a paediatric neuroradiologist using the Rotterdam CT score [13]. The subcategories of the score were also analysed separately for all patients. Postoperative recovery was estimated retrospectively from the patient records using the extended Glasgow outcome scale (GOSE) for all patients [14]. Good recovery was defined as GOSE ≥ 7, and poor as GOSE < 7. Return to school, pupil status and initial Glasgow Coma Scale (GCS) data were available for 23/24 (96%) patients.

We obtained preoperative ICP data from the intensive care unit database of the Oulu University Hospital. Reliable ICP data was available for 7/24 patients and not available for 17/24 patients. Preoperative ICP data was missing in 6 patients since they underwent DC immediately after admission. Additionally, the ICP of 5 patients had been measured during intermittent CSF drainage, thus rendering the measurements unreliable, and high-frequency ICP recording data was missing for 6 patients. The size of the bone defect was measured from the lateral two-dimensional scout CT image, available for 23/24 (96%) patients. Complications following DC were defined as surgical site infection (SSI) requiring reoperation and antibiotics; extracranial hematomas and subcutaneous CSF collections requiring percutaneous tapping and/or reoperation; symptomatic intracerebral hematomas; and hydrocephalus requiring CSF shunting. Additionally, data on smoking and the use of intoxicants was collected retrospectively. Epidemiological data was obtained from Statistics Finland [15]. To ensure no patients were missed in the primary database search, the OUH organ donor service was queried to identify any paediatric patients that had been evaluated for organ donors.

The present study was approved by the medical director of the Oulu University Hospital and the ethical review board of the Northern Ostrobothnia hospital district (decision #111/2015). The study was conducted in accordance with the declaration of Helsinki.

### Statistical analysis

Statistical analyses were conducted with the IBM Statistical Package for the Social Sciences (v. 23, IBM Corp., Armonk, NY, USA). Two-sided Fisher’s exact test was used for the comparison of categorical variables, and the one-way analysis of variance and Mann–Whitney *U* tests for continuous variables. Continuous variables were summarised as means with standard deviation (SD) and range, or medians with interquartile range (IQR). A two-tailed *p* value of < 0.05 was considered statistically significant.

### Clinical protocol

**Fig. 1:**
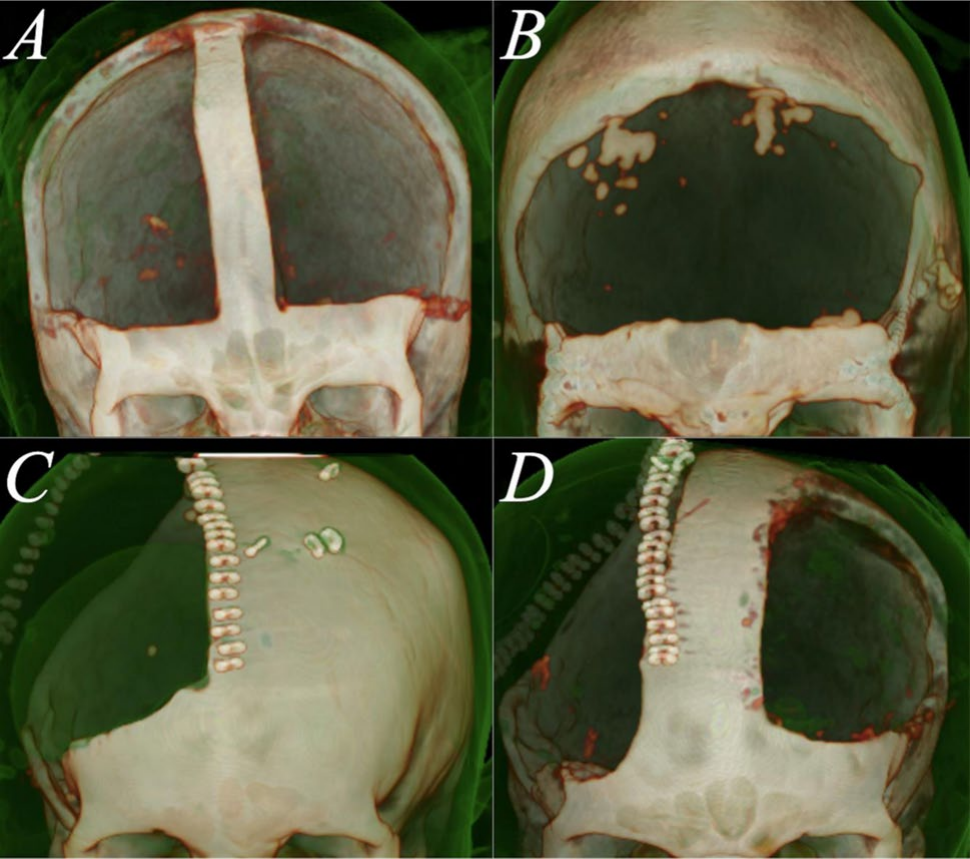
Three-dimensional computed tomography reconstructions of the four types of decompressive craniectomy performed in our centre. **A** depicts a bifrontal two-flap craniectomy, **B** a bifrontal one-flap craniectomy, **C** a unilateral frontotemporoparietal hemicraniectomy and **D** a bilateral hemicraniectomy. In **B**, dural osteogenesis is seen at the craniectomy defect site (scan taken 2 months after DC)

The stepwise treatment protocol of increased ICP at our centre aimed to maintain an ICP of < 20 mmHg similar to the RESCUEicp study by Hutchinson et al. [1]: the initial step comprised head elevation, sedation and analgesia along with normoglycemia, normothermia, normoxemia and the administration of intravenous hyperosmolar solutions. An ICP probe was placed if the patient was unconscious or sedated and intubated. Next, if ICP was not controlled by these measures, sedation was deepened and a ventriculostomy was placed if necessary. Hyperventilation and in some cases mild hypothermia were used if required as a rescue manoeuvre. Barbiturates were not routinely administered, but some patients received thiopental boluses. Finally, if ICP persisted above 20 mmHg, DC was conducted. Four types of craniectomy were used: bifrontal craniectomy with one or two bone flaps, and unilateral or bilateral frontotemporoparietal hemicraniectomy (Fig. 1). The treatment protocol remained unchanged during the study period. The present cohort also includes patients who underwent primary DC, which was conducted upon clinical requirement immediately after admission to OUH.

## Results

### Decompressive craniectomy

In total, 24 patients underwent DC at a median age of 16.0 years (IQR 1.2, range 7.5 to 17.7). The mean follow-up time was 4.2 years (SD 3.6, range 0 days to 10.3 years). The primary diagnoses were TBI in 20 cases (83%), ischemic stroke in two (8%), encephalitis in one (4%) and spontaneous intracranial haemorrhage in one (4%). The median initial GCS was 5.0 (IQR 5.0). Thirteen (65%) of the TBI cases were due to motor vehicle accidents. In TBI patients, the mean Rotterdam CT score in the primary CT scan was 3.0 (SD 1.2, range 1 to 5). DC was conducted at mean 1.6 days (SD 1.6, range 0 to 7 days) after the primary insult—six (25%) craniectomies were primary and 18 (75%) secondary/delayed. The mean size of the craniectomy was 103 cm^2^ (SD 19, range 76 to 147 cm^2^). Six (25%) of the craniectomies were bifrontal one-flap, five (21%) bifrontal two-flap, 12 (50%) unilateral hemicraniectomies and one (4%) bilateral hemicraniectomy (Fig. 1). Baseline characteristics of the DC patients are reported in more detail in Table 1. All the patients with reliable preoperative ICP data (*n* = 7) had a maximum ICP of > 25 mmHg, and the ICPs remained > 20 mmHg for mean 3.4 h (SD 3.0, range 0.24 to 7.95 h) during the last 8 h before DC. See Supplementary Fig. 1 and Supplementary Table 1 for a description of preoperative ICP courses.

**Table 1:** Baseline data stratified by complications of the 21 patients who underwent decompressive craniectomy and survived. Statistical analysis conducted with Fisher’s exact test and one-way analysis of variance

Characteristic	Any complication (*n* = 11)	No complications (*n* = 10)	*p* value
Mean age at craniectomy, years (SD)	15.9 (1.1)	15.5 (2.9)	0.68
Sex			0.66
Male, *n* (%)	8 (73)	6 (60)	
Female, *n* (%)	3 (27)	4 (40)	
Primary diagnosis			0.99
Traumatic brain injury, *n* (%)	9 (82)	8 (80)	
Other, *n* (%)	2 (18)	2 (20)	
Craniectomy site			0.27
Bifrontal, *n* (%)	4 (36)	6 (60)	
Hemicraniectomy, *n* (%)	7 (64)	4 (40)	
EVD or lumbar drain			0.66
Yes, *n* (%)	6 (55)	7 (70)	
No, *n* (%)	5 (45)	3 (30)	
Intoxicant abuse			0.31
Yes, *n* (%)	1 (9)	3 (30)	
No, *n* (%)	10 (91)	7 (70)	
Smoking			0.99
Yes, *n* (%)	1 (9)	1 (10)	
No, *n* (%)	10 (91)	9 (90)	
Mean craniectomy size, cm^2^ (SD)	107 (20)	99 (19)	0.38

*SD* standard deviation, *EVD* external ventricular drain

Three patients (12.5%), all males with polytrauma and TBI, died. One patient died intraoperatively due to excessive brain herniation through the craniectomy bone defect. Two patients died after DC at the intensive care unit. One of these patients developed intractable cerebral swelling during the DC procedure despite maximal treatment and consequently died at the intensive care unit shortly after the procedure. Another patient died due to neurogenic cardiorespiratory failure at the intensive care unit. Notably, all mortality occurred within the first postoperative day after DC. Ability to continue school studies following survival and favourable recovery were similar in patients undergoing primary and secondary DC (*p* = 0.99 and 0.64, respectively).

Of the 21 patients who survived, 11 (52%) developed complications following DC, but only four (19%) required surgery due to the complications. No risk factors for DC complications were found (Table 1). Primary complications are reported in Table 2. Three patients had multiple complications. One had an intracerebral hematoma following external ventricular drain (EVD) placement, SSI and an intracerebral abscess at the site of the hematoma, and she eventually developed shunt-dependent communicating hydrocephalus. Primarily, this patient had systemic leukaemia and developed an intracerebral hematoma due to thrombocytopenia and underwent secondary DC. One patient also had an SSI and later developed shunt-dependent communicating hydrocephalus. The third patient had a subcutaneous CSF collection that required percutaneous tapping and later developed communicating hydrocephalus requiring CSF shunting after his second cranioplasty. No other patients had multiple complications.

**Table 2:** Primary complications of the 21 surviving patients after decompressive craniectomy

Complication	Number (% of total)	Mean days from DC to complication (range)
Surgical site infection	1 (5)	16
Shunt-requiring CSF disorder^a^	4 (19)	98 (34 to 227)
Subcutaneous CSF collection	6 (29)	13 (8 to 21)
Total	11 (52)	40 (8 to 227)

*DC* decompressive craniectomy, *CSF* cerebrospinal fluid

^a^Three patients developed shunt-dependent communicating hydrocephalus after decompressive craniectomy, before cranioplasty. One patient developed shunt-dependent hydrocephalus following secondary cranioplasty 227 days after craniectomy

### Long-term follow-up

**Fig. 2:**
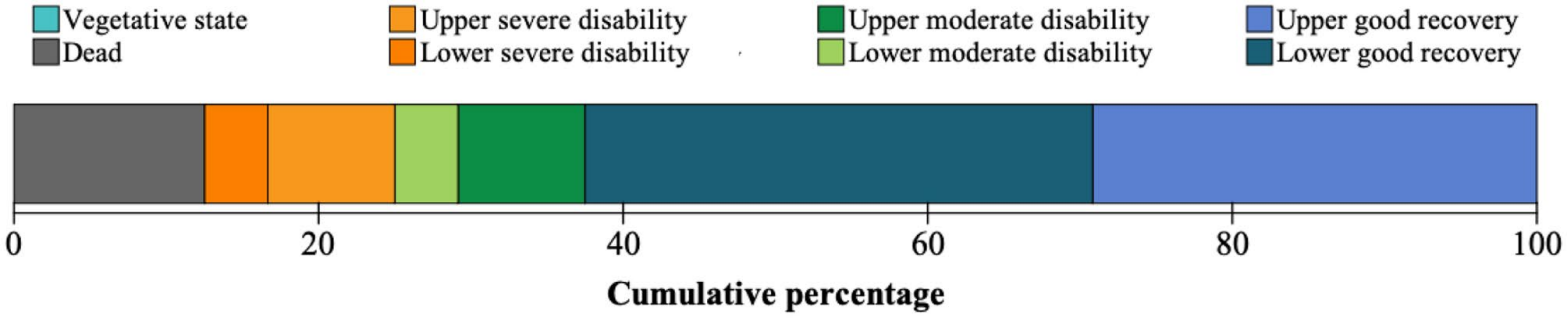
Extended Glasgow Outcome Scale scores at the last follow-up dates of decompressive craniectomy patients (*n* = 24)

Measured at the last follow-up dates, the mean GOSE score of our patients was 6.0 (SD 2.4). Fifteen (63%) patients had made a good recovery (GOSE ≥ 7) during the follow-up time. Of the surviving 21 patients, eighteen (86%) had returned to school as of the last follow-up date. The GOSE scores are depicted in Fig. 2. More detailed information is provided in Supplementary Table 2.

Patients with shunt-requiring hydrocephalus were less likely to have made a good recovery or returned to school as of the last follow-up date, and TBI patients had made good recoveries more often than patients with other primary diagnoses (Table 3). Age, initial GCS, gender, the placement of an EVD or a lumbar drain, time between brain injury and DC were not associated with good or bad recovery or the patient’s ability to return to school (Table 3). Preoperative pupil status was not associated with the recovery parameters (Table 3), but it demonstrated borderline correlation with mortality (*p* = 0.05). Of note, one patient with preoperative bilateral mydriasis survived and made a favourable recovery.

**Table 3:** Analysis of the factors associated with return to school in surviving patients (*n* = 21) and good recovery in all patients (*n* = 24). Statistical assessment conducted using Fisher’s exact test, one-way analysis of variance and Mann–Whitney *U* test as appropriate

Variable	Return to school ^a^		*p* value	GOSE 7 or 8		*p* value
	Yes (*n* = 18)	No (*n* = 2)		Yes (*n* = 15)	No (*n* = 9)	
Mean age at craniectomy, years (SD)	15.6 (2.2)	15.3 (0.2)	0.83	16.1 (1.0)	14.3 (3.8)	0.21
Median initial GCS (IQR)	6.0 (6.0)	6.5 (N/A)	0.75	4.5 (4.0)	6.0 (6.0)	0.31
Preoperative mydriasis			0.52			0.69
No mydriasis	13 (72)	1 (50)		11 (73)	5 (56)	
Unilateral	4 (22)	1 (50)		3 (20)	2 (22)	
Bilateral	1 (6)	0 (0)		1 (7)	2 (22)	
Mean days from primary insult to DC (SD)	1.3 (1.2)	2.5 (0.7)	0.20	1.8 (1.9)	1.2 (1.1)	0.42
Mean months from DC to cranioplasty (SD) ^b^	3.6 (2.5)	2.2 (0.0)	0.61	3.2 (1.4)	4.3 (4.5)	0.43
Sex			0.99			0.99
Male, *n* (%)	12 (67)	1 (50)		11 (73)	6 (67)	
Female, *n* (%)	6 (33)	1 (50)		4 (27)	3 (33)	
Traumatic brain injury			0.37			0.01*
Yes, *n* (%)	15 (83)	1 (50)		15 (100)	5 (56)	
No, *n* (%)	3 (17)	1 (50)		0 (0)	4 (44)	
Shunt-requiring hydrocephalus			0.03*			0.01*
Yes, *n* (%)	2 (11)	2 (100)		0 (0)	4 (44)	
No, *n* (%)	16 (89)	0 (0)		15 (100)	5 (56)	
EVD or lumbar drain in acute phase			0.50			0.99
Yes, *n* (%)	10 (56)	2 (100)		9 (60)	5 (56)	
No, *n* (%)	8 (44)	0 (0)		6 (40)	4 (44)	

*GOSE* extended Glasgow Outcome Scale, *DC* decompressive craniectomy, *SD* standard deviation, *EVD* external ventricular drain

^a^Return to school data available for 20/21 living patients

^b^One of the two patients that did not return to did not undergo cranioplasty

^*^p < 0.05 for statistically significant difference between groups

In patients with TBI, the Rotterdam CT score in the primary CT scan was not associated with the outcome parameters (Table 4). As shown in Table 5, absent basal cisterns in the primary CT scan were associated with mortality and the patient not returning to school, but not with poor recovery. Importantly, among surviving patients, radiological basal cistern status was not associated with GOSE or inability to return to school (p = 0.81, p = 0.58, respectively).

**Table 4:** Effects of the Rotterdam computed tomography score on the patient’s ability to return to school, recovery and mortality in patients with traumatic brain injury (*n* = 20). Statistical analysis conducted with one-way analysis of variance

Variable	Return to school ^a^		*p* value	GOSE 7 or 8		*p* value	Death		*p* value ^b^
	Yes (*n* = 15)	No (*n* = 4)		Yes (*n* = 15)	No (*n* = 5)		Yes (*n* = 3)	No (*n* = 17)	
Mean Rotterdam CT score (SD)	2.9 (1.3)	3.5 (1.0)	0.43	2.9 (1.3)	3.4 (0.9)	0.41	4.0 (0.0)	2.8 (1.2)	0.13

*GOSE* extended Glasgow Outcome Scale, *CT* computed tomography, *SD* standard deviation

^a^Return to school data available for 19/20 traumatic brain injury patients

^b^Zero variance in one group and violated variance homogeneity assumption

**Table 5:** Effects of radiological variables on long-term recovery and mortality in decompressive craniectomy patients (*n* = 24). Statistical analysis conducted with Fisher's exact test

Variable	Return to school ^a^		*p* value	GOSE 7 or 8		*p* value	Death		p value
	Yes (*n* = 18)	No (*n* = 5)		Yes (*n* = 15)	No (*n* = 9)		Yes (*n* = 3)	No (*n* = 21)	
Basal cisterns			0.04*			0.48			0.005*
Normal, *n* (%)	8 (44)	2 (40)		8 (53)	3 (33)		0 (0)	11 (52)	
Compressed, *n* (%)	8 (44)	0 (0)		5 (33)	3 (33)		0 (0)	8 (38)	
Absent, *n* (%)	2 (11)	3 (60)		2 (13)	3 (33)		3 (100)	2 (10)	
Midline shift			0.99			0.99			0.52
≤5 mm, *n* (%)	14 (78)	4 (80)		12 (80)	7 (78)		2 (66)	17 (81)	
>5 mm, *n* (%)	4 (22)	1 (20)		3 (20)	2 (22)		1 (33)	4 (19)	
Epidural mass lesion			0.54			0.26			0.99
Present, *n* (%)	4 (22)	0 (0)		4 (27)	0 (0)		0 (0)	4 (19)	
Absent, *n* (%)	14 (78)	5 (100)		11 (73)	9 (100)		3 (100)	17 (81)	
Intra-ventricular blood or tSAH			0.62			0.36			0.99
Present, *n* (%)	13 (72)	3 (60)		12 (80)	5 (55)		2 (67)	15 (71)	
Absent, *n* (%)	5 (28)	2 (40)		3 (20)	4 (44)		1 (33)	6 (29)	

*GOSE* extended Glasgow outcome scale, *tSAH* traumatic subarachnoid haemorrhage

^a^Return to school data was available for 23/24 patients

^*^p < 0.05 for statistically significant difference between groups

### Cranioplasty

All but one (95%) of the surviving patients underwent cranioplasty at mean 3.5 months (SD 2.4, range 1.3 to 12.2) after DC. One of the cranioplasties was performed in another university hospital. Twelve (60%) of the primary cranioplasties were conducted using an autologous cryopreserved bone flap, and eight with patient specific implants. In seven cases (35%), fibre-reinforced composite bioglass (FRC-BG) implants were used, and one patient (5%) received a polymethylmethacrylate (PMMA) implant. Five (25%) patients developed complications after cranioplasty with four (20%) requiring re-cranioplasty. These re-do surgeries were done in three cases (15%) due to SSIs and in one (8% of autologous cranioplasties) due to clinically significant bone flap resorption. SSI developed in one patient with autologous, one with PMMA and one with FRC-BG implants. The only patient that had conservatively treated complication had an extracranial hematoma that was treated by repeated tapping. Three (75%) of the secondary cranioplasties were conducted with an FRC-BG implant and one (25%) with a PMMA implant.

The material used in the primary cranioplasty did not affect complication rates (*p* = 0.33). Timing of the cranioplasty procedure did not affect the patients’ recovery or their ability to return to school (Table 3). Following autologous cranioplasty, the mean Oulu resorption score was 3.7 (SD 3.4, range 0 to 9). When three patients with radiological follow-up times of < 7 days were excluded, the mean ORS was 5.1 (SD 2.9, range 0 to 9) with a mean follow-up time of 31.6 months (SD 30.6, range 4 to 80 months).

### Epidemiological data

Between the years 2009 and 2019, the paediatric (under 18 years of age) catchment population of the OUH ranged between 156 347 and 165 669 children with a mean of 162 344 children [15]. Thus, the mean yearly prevalence of paediatric brain injury severe enough to require DC was 1.34 per 100 000 children during the study period. Six additional paediatric patients were identified from the OUH’s organ donor registry from between 2009 and 2019. Four of these patients had sustained high-energy TBI and polytrauma that was deemed unsurvivable and inoperable, one patient had an anoxic brain injury due to acute viral respiratory distress syndrome, and one a viral encephalitis with sinus thrombosis. In total, the mean yearly prevalence of very severe brain injury was 1.57 per 100 000 children.

## Discussion

We presented a series of consecutive paediatric patients who underwent DC due to intractably increased ICP in the Oulu University Hospital during a time period of 11 years. The Oulu University Hospital serves geographically more than half of Finland with a catchment population of 740 000. As in previous studies [4, 11], the most common indication for DC was TBI comprising 83% of all cases in the present study. Most of the brain injuries arose due to motor vehicle accidents, and the mean age of the present study population was 15.4 years. This is in line with epidemiological reports on the aetiology and incidence of paediatric TBI [16].

According to our data, the mean yearly prevalence of paediatric brain injury severe enough to require DC was approximately 1.34 per 100 000 children between the years 2009 and 2019. During the same time interval, 90 patients aged 18–65 years underwent DC at our institution (unpublished data, A. Lammi, 2021). Thus, the mean yearly prevalence of adult brain injury severe enough to require DC was 1.84 per 100 000 adult inhabitants. The present DC cohort predominantly consists of adolescent patients as the youngest patient was 7.5 years old. Thus, in the Northern Finland, brain injury requiring DC is rare especially among toddlers.

### Clinical outcome after decompressive craniectomy

As expected, the present cohort predominantly consisted of TBI patients. Mortality was low, 12.5%, which is favourable compared to previous reports [8, 17, 18]. All the patients that died had undergone high-energy motor vehicle accidents. Notably, all three fatalities occurred within 24 h from the DC procedure: one patient died during the DC procedure, and two during the first postoperative day at the intensive care unit. Even though the initial prognosis appeared poor due to severe TBI and polytrauma, these patients were operated—a reflection of our institution’s aggressive treatment protocol of paediatric patients with life-threatening intracranial injury. One could speculate that hopeless cases were operated since one death occurred during the DC procedure. However, no survivors were in vegetative state (Fig. 2). On the contrary, almost two-thirds of the surviving patients had made a good recovery (GOSE ≥ 7), and 90% had eventually returned to normal school with or without support. Of note, the present cohort mainly comprised adolescent patients, which may influence the observed rate of favourable outcome as younger age is traditionally taken as a predictor of poor outcome after paediatric TBI [19–21]. In the setting of DC, however, the effect of age is more unclear, as contradicting associations between age and mortality or recovery have been reported [3, 4, 22, 23].

Though the mean Rotterdam CT score in the primary CT scan was relatively low (3.0), the median initial GCS was 5.0, and eight (33%) of the patients had mydriasis, of which three were bilateral. Generally a contraindication for DC, bilateral mydriasis in our population developed whilst already in the operating room for DC. Though bilateral mydriasis expectedly predicted death, one patient with bilaterally mydriatic pupils survived with upper good recovery and was able to continue school. This is probably due to very short delay from the development of mydriasis to the DC. All the patients with reliable preoperative ICP data had a maximum ICP > 25 mmHg (Supplementary Fig. 1 and Supplementary Table 1). ICP doses varied notably between the patients, which may affect outcomes, but further analyses would require improved ICP data availability. Based on the present series, the long-term prognosis of paediatric patients after successful DC appears favourable especially in the setting of TBI, as suggested by previous research [4, 23].

Furthermore, the catchment area of the OUH is rurally dominated and logistical parameters such as transfer times may differ from those in more densely populated neurosurgical catchment areas. The effect of this probable difference requires further research, especially as there is paucity of data on primary DC in general [24]. The present study included six primary (25%) and 18 secondary (‘delayed’) (75%) craniectomies. The authors found no correlation between the time from the primary insult to DC and the recovery status as measured with GOSE or the patient’s ability to return to school, thus highlighting the importance of clinical judgement before DC, as refractorily high ICP develops with varying delay from the primary injury. The mean time of 1.6 days from the insult to DC in the present study corresponds to those published earlier [3, 22, 23].

The Rotterdam CT score has been validated as a prognostic measure in adult TBI populations [13], and to some degree in paediatric TBI populations [25]. However, in the present study, the Rotterdam CT score in the primary CT scan was not associated with the patient’s ability to return to school, poor recovery (GOSE < 7) or death. Obliteration of the basal cisterns was associated with increased mortality. Encouragingly, absent basal cisterns were not associated with poor recovery or inability to continue school if the patient survived DC. Thus, DC appears to result in satisfactory outcomes even in clinically and radiologically severe brain injury among paediatric patients. Results supporting these findings have been described previously in the literature [4, 23].

### Complications after decompressive craniectomy

Fifty-two percent of our patients had complications after DC. Two-thirds of the primary complications were subcutaneous CSF collections. Correspondingly, only four (19%) patients required surgery due to DC complications. Three (13%) patients required surgical intervention due to SSI after DC, and four ventriculoperitoneal shunts had to be placed during the follow-up period. In this paediatric cohort, the timing of DC complications followed the sequential incidence pattern of DC complications proposed in previous studies with predominantly adult populations (Table 2) [26–28].

Subcutaneous CSF collections requiring percutaneous tapping were considered complications in the present study. Occurring in 30% of our DC patients and treated predominantly by tapping the collection, they comprised the majority of DC complications. Similar numbers have been reported in previous studies [29]. It must be brought to question whether these collections represent a complication or an inevitable side effect of the DC procedure. They probably arise due to the surgical technique of DC as the dura is intentionally left open and the dural defect is loosely covered with artificial duroplasty materials. This creates additional space for the oedematous brain to expand in, but after the subsidence of the oedema, also leaves dead space for the CSF to fill. Indeed, the incidence of subcutaneous CSF collections was 15% (2/13) in patients who had a ventriculostomy or a lumbar drain in place after DC, as compared to 63% (5/8) in those who did not, but statistical significance was not reached (*p* = 0.06). Some of these patients with CSF collections may also have had temporary CSF circulation disorders that resolved spontaneously without further CSF diversion procedures.

Severe TBI and DC are associated with a high incidence of post-traumatic hydrocephalus [18, 30]. In total, four (19%) of the surviving patients developed shunt-dependent hydrocephalus: three before cranioplasty, and one patient after their secondary cranioplasty, which was conducted due to SSI of the primary cranioplasty. Hydrocephalus after DC may arise due to the primary insult itself, but the altered CSF dynamics and postoperative complications after DC may also predispose patients to hydrocephalus. Shunt-dependent hydrocephalus seems to be a negative prognostic factor following DC: all of our patients with CSF shunts in the present study achieved poor recovery measured by GOSE and returned to school less often than those with no shunts. Intuitively, hydrocephalus may indicate more severe TBI. In a recent retrospective report enrolling 91 583 TBI patients aged under 22 years, the incidence of hydrocephalus was greater in patients with more severe TBI [30], which may explain the poorer prognosis of the shunt-dependent hydrocephalic patients in the present study.

We assume that hydrocephalus is a complication of the primary insult whether it be TBI, stroke or infection. On the other hand, it could be speculated that large bone defects interfere with normal CSF circulation causing secondary hydrocephalus as a complication of DC [31]. Indeed, in the aforementioned study [30], cranioplasty within 30 days after DC was associated with a decreased incidence of hydrocephalus. As it was impossible to differentiate whether CSF-related events were complications of the primary insult or DC, we decided to count them as DC complications in the present study.

### Cranioplasty

Twenty (95%) of the DC patients underwent subsequent cranioplasty after a mean period of 3.5 months from the DC. No association between the implant material and complications or the timing of cranioplasty and the outcome parameters was found. Complications requiring re-cranioplasty occurred in 20% of the patients after cranioplasty. This is lower than previous reports especially in terms of bone flap resorption [9–11]. The rate of clinically significant bone flap resorption in our predominantly adolescent patient cohort was 8% among patients with autologous cranioplasty. Indeed, the incidence of resorption seems to decrease with increasing age within the paediatric population. A BFR prevalence of 100% was reported in patients aged less than a year, and 70% in those younger than 2.5 years [32, 33] with larger studies reporting BFR rates of 16.5–57.5% in general paediatric populations [7, 9–11, 34]. Low BFR is probably due to the high proportion of adolescents in our study cohort unlike in the series cited above.

Anyhow, radiological bone flap resorption changes were detected in 7/8 (88%) of the patients with sufficient follow-up time. The mean ORS was 5.1, which is slightly lower than in a study by Beez on a slightly younger patient cohort [8]. Importantly, the temporal progression of the bone flap resorption process is currently unknown in the literature—anecdotal evidence suggests that the process may stabilise during the first 2 postoperative years in adults [35–37].

### Strengths and weaknesses

First, the findings of the present study are to be interpreted with regard to the small sample size of the present study. As such, the effect of chance on the results may be notable. The study was conducted in a retrospective manner and thus it is subject to the biases associated with such setting. Nevertheless, we managed to describe clinically relevant recovery predictors for paediatric DC patients. Furthermore, no patients were lost to follow-up. The decision to conduct a DC was based on the stepwise treatment protocol of increased ICP and thus the present cohort represents a selected subpopulation of paediatric TBI patients—a selection bias is inherent in the epidemiological calculations.

Customary to the Finnish healthcare system, all acute neurosurgical care and follow-up, including the treatment of complications, is provided only by the neurosurgical units of five University Hospitals. All neurosurgical patients from the Northern Finland are treated in the Oulu University Hospital. Long-term follow-up data of two patients who were not residents of the Oulu University Hospital district, but got injured during vacation, was gathered from their local neurosurgical units where they were referred after stabilisation of their condition. Therefore, on the population level, the present study accurately represented the epidemiology of very severe paediatric brain injury and DC in our region.

## Conclusions

According to our findings, DC is a life-saving procedure with the majority of paediatric patients making a favourable recovery despite clinically and radiologically severe primary insult. Especially, good outcome was achieved in patients suffering from traumatic brain injury. Almost two-thirds of the surviving patients had made a good recovery, and 90% had returned to school. None were in vegetative state on the last follow-up date. Further study in a multicentre setting is warranted.

## Supplementary Information

Below is the link to the electronic supplementary material.

Supplementary file1 (DOCX 197 kb)

## Data Availability

Original materials are available for review upon reasonable request to the corresponding author.

## Code availability

No custom code was used and the relevant software were described in the manuscript.
