# Supplementary material for: Favourable long-term recovery after decompressive craniectomy: the Northern Finland experience with a predominantly adolescent patient cohort
Source: Childs Nerv Syst. 2022 Jun 24;38(9):1763–72. doi: 10.1007/s00381-022-05568-7 (PMC9463249; doi:10.1007/s00381-022-05568-7)
Supplement: Supplementary file 1 — Supplementary file1 (DOCX 197 kb) [file 381_2022_5568_MOESM1_ESM.docx]

**Supplementary information**

**Supplementary Figure 1.** Intracranial pressure (ICP) profiles of 7 patients who underwent secondary or delayed decompressive craniectomy. Each line represents individual patients. Reliable ICP data was not available for 17 patients: the ICP of 5 patients had been measured during intermittent CSF drainage thus rendering the measurements unreliable; 6 patients underwent a primary decompressive craniectomy; and high-frequency ICP recording data was missing for 6 patients.

| **Supplementary Table 1.** Preoperative ICP parameters of 7 paediatric patients who underwent decompressive craniectomy due to traumatic brain injury. All the patients made a favorable recovery and were able to continue school. As such, detailed outcome analyses are precluded. | | | | | |
| --- | --- | --- | --- | --- | --- |
| Patient | Mean ICP (mmHg) | Time of ICP observation (h) | Maximum ICP (mmHg) | ICP dose* [h (%)] |  |
| 1 | 19.8 | 7.7 | 40.0 | 3.23 (40.4) |  |
| 2 | 21.4 | 13.0 | 49.6 | 6.58 (82.2) |  |
| 3 | 19.8 | 8.9 | 43.3 | 4.20 (52.5) |  |
| 5 | 22.6 | 34.2 | 43.1 | 7.95 (99.4) |  |
| 8 | 14.3 | 26.9 | 38.8 | 1.33 (16.6) |  |
| 10 | 14.6 | 12.2 | 28.6 | 0.24 (3.0) |  |
| 18 | 15.6 | 52.5 | 29.4 | 0.27 (3.3) |  |
| ICP = intracranial pressure | | | | |  |
| * ICP dose calculated as hours the ICP exceeded 20 mmHg during the last 8 hours before decompressive craniectomy and the respective percentage. | | | | |  |

|  | | **Supplementary Table 2.** Data on decompressive craniectomy patients' primary insult, recovery and follow-up time. | | | | | | | | |  |  |
| --- | --- | --- | --- | --- | --- | --- | --- | --- | --- | --- | --- | --- |
| Patient | Age | | Primary insult | GCS | GOSE | Return to school | Follow-up time (yrs) | Brief status description | |  |  |  |
| 1 | 16 | | Car accident | 6 | 8 | Yes | 6.2 | Good recovery, graduated to a manual job. | |  |  |  |
| 2 | 17 | | Moped crash | 5 | 7 | Yes | 10.2 | Memory problems, symptomatic epilepsy, but graduated to a manual job. | |  |  |  |
| 3 | 15 | | Moped crash | 12 | 7 | Yes | 10.3 | Memory problems, unilateral hearing loss, but returned to school. | |  |  |  |
| 4 | 15 | | Moped crash | 5 | 4 | No | 10.3 | Shunt-dependent hydrocephalus, epilepsy, spastic tetraparesis treated with baclofen, extensive cognitive deficits, but able to walk and live at home. | |  |  |  |
| 5 | 15 | | Collapsed and hit head | 15 | 8 | Yes | 7.8 | Good recovery, mild psychological symptoms that subsided, graduated. | |  |  |  |
| 6 | 16 | | Collapsed and hit head | 6 | 8 | Yes | 7.7 | Good recovery, neuropsychologically assessed as gifted. | |  |  |  |
| 7 | 16 | | Moped crash | 3 | 7 | Yes | 7.8 | Good recovery, able to walk, talk and drive a car, undergoing rehabilitation. | |  |  |  |
| 8 | 16 | | Hit by car (no helmet) | 8 | 7 | Yes | 3.4 | Memory problems, double images due to posttraumatic strabismus, headaches, but independent. | |  |  |  |
| 9 | 7 | | Encephalitis, thalassemia | 7 | 6 | Yes | 1.4 | Good recovery, concentration difficulty, behavioural changes, fluctuating memory loss, requires support in stairs, nocturia, but able to walk and talk normally. | |  |  |  |
| 10 | 15 | | Skiing accident | 9 | 7 | Yes | 3.1 | Good recovery, loss of olfaction, but able to walk and talk normally, no neurological deficit. | |  |  |  |
| 11 | 17 | | Fell from third floor | 6 | 4 | Yes | 3.3 | Lives on their own, but requires the help of a personal assistant in activities of daily living for a couple of hours daily. | |  |  |  |
| 12 | 16 | | Moped crash | 3 | 8 | Yes | 1.5 | Good recovery, neuropsychologically assessed as normal, continued high school. | |  |  |  |
| 13 | 13 | | Bicycle accident (no helmet) | 4 | 8 | Yes | 1.6 | Good recovery, very mild cognitive symptoms, continued education. | |  |  |  |
| 14 | 16 | | Car accident | 5 | 7 | Yes | 4.1 | Good recovery, about to begin supported education to a manual job. | |  |  |  |
| 15 | 16 | | Arterial stroke | 13 | 5 | Yes | 2.5 | Hemiparesis, field of view deficit, cognitive symptoms, but continued high school studies. Independent, but functions slower than before. | |  |  |  |
| 16 | 16 | | Venous stroke | 10 | N/A | Yes | 3.1 | Hemiparesis mostly affecting the non-dominant hand, able to walk, continued high school. | |  |  |  |
| 17 | 15 | | Moped crash | 3 | 8 | Yes | 1.6 | Good recovery, mild cognitive symptoms, good memory. | |  |  |  |
| 18 | 15 | | Hit a wall running | 3 | 8 | Yes | 0.7 | Good recovery, no neuropsychological deficits. A small field of view deficit. Does not require any special rehabilitation. | |  |  |  |
| 19 | 15 | | Moped crash | 3 | 7 | Yes | 0.3 | Good recovery, returned to school. Blind in one eye. | |  |  |  |
| 20 | 15 | | Leukaemia, ICH | 5 | 3 | No | 10.2 | Hemiparesis, requires a wheelchair and at least supervision whilst moving. Conversates normally despite cognitive deficits. | |  |  |  |
| 21 | 17 | | Skiing accident | 3 | N/A | N/A | 0.3 | Transferred to another hospital for cranioplasty and rehabilitation. | |  |  |  |
| 22 | 16 | | Hit by a train | 3 | 1 | No | 0.0 | Dead. | |  |  |  |
| 23 | 16 | | Moped crash | 4 | 1 | No | 0.0 | Dead. | |  |  |  |
| 24 | 7 | | Hit by a car | 3 | 1 | No | 0.0 | Dead. | |  |  |  |
|  | | GOSE=extended Glasgow Outcome Scale, N/A=not available, ICH = intracerebral hemorrhage | | | | | | |  |  |  |  |
